# Understanding the extent to which PROMs and PREMs used with older people with severe frailty capture their multidimensional needs: A scoping review

**DOI:** 10.1177/02692163231223089

**Published:** 2024-01-24

**Authors:** Faith D Howard, Richard Green, Jenny Harris, Joy Ross, Caroline Nicholson

**Affiliations:** 1 Department of Health Sciences, University of Surrey, Guildford, UK; 2 St Christopher’s Hospice, London, UK

**Keywords:** Frailty, aged, patient reported outcome measures, palliative care, questionnaire, patient centred care

## Abstract

**Background:**

Older people with severe frailty are nearing the end of life but their needs are often unknown and unmet. Systematic ways to capture and measure the needs of this group are required. Patient reported Outcome Measures (PROMs) & Patient reported Experience Measures (PREMs) are possible tools to assist this.

**Aim:**

To establish whether, and in what ways, the needs of older people living with severe frailty are represented within existing PROMs and PREMs and to examine the extent to which the measures have been validated with this patient group.

**Design:**

The scoping review follows the method of Arksey and O’Malley.

**Results:**

Seventeen papers from 9 countries meeting the inclusion criteria and 18 multi-dimensional measures were identified: 17 PROMs, and 1 PROM with PREM elements. Seven out of the 18 measures had evidence of being tested for validity with those with frailty. No measure was developed specifically for a frail population. Using the adapted framework of palliative need, five measures covered all five domains of palliative need (IPOS, ICECAP-SCM, PDI, WHOQOL-BREF, WHOQOL-OLD). The coverage of items within the domains varied between the measures.

**Conclusion:**

Existing PROMs and PREMs are not well designed for what we know about the needs of older people with severe frailty. Future research should firstly focus on adapting and validating the existing measures to ensure they are fit for purpose, and secondly on developing a better understanding of how measures are used to deliver/better person-centred care.

**What is already known about the topic?**

- Older people with severe frailty have end-of-life needs.
- Patient reported outcome measures and Patient reported experience measures are useful patient-centred clinical tools to capture and measure needs systematically.
- There is good evidence that the use of PROMs and PREMs within end-of-life care improves outcomes for patients.

**What this paper adds?**

- A limited number of PROMs and PREMs have been validated with older people with frailty.
- There is a disparity between item coverage within PROMs and PREMs being used, particularly within the practical and spiritual domains and the known end-of-life needs of older people with severe frailty.

**Implications for practice, theory, or policy**

- Research is required to understand how measures are implemented and used in practice to capture what matters most at an individual level for older people with frailty.
- Providers need to consider how PROMs and PREMs may be used together and the practical factors that may influence how this is achieved.
- Future research should focus on adapting and validating existing PROMs and PREMs to ensure they are fit for purpose with this group.

## Background

As we live longer and with more complex needs, frailty has become a growing global priority.^1^ Older people with severe frailty are nearing the end of their lives, and there is growing evidence that what matters most to this group goes beyond just their physical health needs. The priorities of older people with frailty are often concerned with quality of life, maintaining and strengthening their relationships, and being involved in conversations about their current and future care.^2^ Despite this, care services are often oriented towards physical health needs, and the available patient-centred tools to identify need have not been developed with this group in mind. What remains under-explored is how such tools are being tailored to identify the individual end-of-life needs of this group, and whether these tools capture and measure what matters most to older people living with frailty.

In part due to the uncertainty that is associated with the disease trajectory of those with frailty, it has been suggested that accessing care should be based on current needs, rather than prognosis.^3^ However, defining need is complex, resulting in numerous definitions and app-roaches to its measurement.^4^ From a sociological perspective, *normative* need can be defined as those set by experts or standards, whereas e*xpressed* need is considered to be those needs vocalised by the individual.^5^ Tools used to assess the need for a palliative and end-of-life care approach (such as the ‘surprise question’) have poor reliability with older people with frailty.^6^ Professionals and providers require alternative ways to capture and measure this group’s end-of-life needs systematically. Patient reported Outcome Measures (PROMs) and Patient reported Experience Measures (PREMs) are possible tools to assist this. These are standardised, validated questionnaires completed by the patient or proxy which aim to capture outcomes and experiences from the patient’s point of view.^7^ PROMs and PREMs can be used to guide clinical assessments of individuals, understand the wider population needs, measure quality of care, and effectiveness of interventions.^8^ Within specialist palliative care, PROMs and PREMs have been developed to measure outcomes that are meaningful to the patient and which improve quality of care.^9–12^ Most individuals with severe frailty, however, will not be seen by palliative care specialists. They will more typically receive care from a spectrum of older adult health and social care providers in a variety of settings, often using different methods of assessing need where PROMs and PREMS may be missing and required. An understanding of the types of self-reported measures in use with this group of patients, and the extent to which these reflect common needs associated with frailty towards the end of life is required in order for providers to deliver meaningful care for this population.

A recent review examined PROMs used with those with frailty within emergency and acute settings^13^ but to the author’s knowledge, no review has addressed this issue in relation to older people with severe frailty nearing the end of life. The limited evidence exploring the priorities and needs of older people with frailty toward the end of life indicate that their needs are multi-dimensional. Needs were found to be related to pain, function, emotional support, maintaining and strengthening relationships, future planning, having access to individualised care, and quality of life.^2,3,14^ The availability of PROMs and PREMs which reflect their multi-dimensional needs is therefore, important to support person centred care. In this review the end-of-life needs and experiences of those with frailty using the conceptual framework of palliative need are considered.^15^ In addition to the four frequently used palliative care domains: physical, psychological, social and spiritual this review incorporates an additional fifth ‘practical’ domain.^2^

The following questions are posed in this review: i) Are the multi-dimensional needs experienced by those with severe frailty conceptualised within Patient reported outcome measures (PROMs) and Patient reported experience measures (PREMs) and, if so, how? And ii) What evidence is there that PROMs and PREMs have been validated with this patient group?

## Design

Arksey and O’Malley’s approach for conducting scoping reviews was used. This review design is used to gain an overview of the literature, to clarify concepts and identify the characteristics that relate to concepts. It can also be used to help identify gaps in the existing literature and critically analyse the overall state of research.^16^ This is particularly useful where the evidence related to the use of PROMs and PREMs for older people living with severe frailty is limited. The Preferred Reporting Items for Systematic Reviews and Meta-Analyses extension for Scoping Reviews (PRISMA-ScR) Checklist was used following guidance by Peters et al.^17^ to facilitate reporting of the scoping review.^18^ See Supplemental Data File 1.

### Search strategy and inclusion criteria

A scoping review allows for an iterative process. The underpinning concept, iterations of the research question and search terms were developed by all authors. An initial search was conducted within MEDLINE and CINAHL to identify relevant papers. Keywords and index terms identified within the titles and abstracts were recorded. These were used to develop the final detailed search strategy which was agreed upon by the authors. See Supplemental Data File 2. Five databases were used for the final full search [(CINAHL (Ebscohost), Medline (Ebscohost), and APA PsychINFO (Ebscohost); ASSIA (ProQuest) and Scopus (Elsevier)]. Scoping reviews allow for the inclusion of grey literature, thus Web of Science was also used, but search terms were only applied to the abstract and title to manage the volume of citations from this database. Four clinicians and three expert researchers within the field of palliative care in the United Kingdom were also consulted to identify measures the search strategy might be expected to identify. The search was limited from 1st January 2012 to the 27th April 2023 to reflect the increasing prominence of frailty in recent years.

As no internationally agreed standard definition for frailty exists, rather than imposing a definition on the search, studies were considered eligible if they provided their own definition of frailty (and/or at least half of the sample were identified as living with frailty). As the aim focused on validation, this review sought to examine how measures were developed and used to capture the needs of the defined population, rather than the identification of studies that used PROMs or PREMs to assess a component of an intervention. Studies were, therefore, eligible if they used multi-dimensional PROMs or PREMs to capture the needs or goals of the participants, if they were validation studies for a multi-dimensional PROM or PREM, or if they described the experience of using multi-dimensional PROMs or PREMs. Studies that used PROMs or PREMs solely to measure the effectiveness of an intervention were excluded.

Hall et al.^19^ found little evidence for the use of established methods to assess prognostication of end-of-life for those with frailty. Studies were, therefore, considered eligible if they were set in any health or social care setting associated with providing care to those towards end-of-life.^13^ Table 1 provides full details of the inclusion criteria applied to the search.

**Table 1.** Inclusion and exclusion criteria.

Category	Inclusion	Exclusion
Older people	More than half the sample is 60 years of age or older with or without cognitive impairment	Less than half the sample is 60 years of age or older OR Information is not available.
Frailty	Any study’s definition of frailty will be accepted OR More than half of the sample are older people with frailty.	More than half the sample is identified as non-frail (including prefrail) OR Details of those with frailty cannot be extracted
End-of-life	Participants are defined as being in the last 1–2 years of life OR Participants living with severe frailty^a^	Participants are not living with severe frailty OR Information not available
Community	The study is within a community setting^b^ OR Transitional care between acute and community settings	The study in severely acute care setting^c^
PROMs or PREMs	Complete multi-dimensional PROMs or PREMs are used to capture need or goals of the participants. OR Validation study for a multi-dimensional PROM or PREM^d^	Measures exclusively completed by professionals (i.e., independent of cognitive function, level of sickness or fatigue) OR Self-reporting measures that have been designed as a diagnostic tool (i.e., identify or measure severity of frailty). OR PROMs or PREMs that have been used to evaluate the effect of an intervention
Relevance	The study is relevant to the research question.	The study is not relevant to the research question.
Year of publication	Papers published between 2012 to 30th May 2022	Papers published prior to 2012
Study design	Empirical studies	Non-empirical studies

a Where the degree of severity of frailty or end-of-life need is not explicit within the paper the following question should be asked: Does the sample population within the study help our understanding of the PROMs and PREMs?.

b These may include primary care, community hospital wards, geriatric day care or clinics, in-patient hospice and hospice at-home services, general practice, community nursing, residential care, nursing homes, adult social care and voluntary sectors used with those with advancing frailty? For example, a setting such as a nursing home might indicate advancing frailty within the sample – Such studies may justify inclusion (even when End-of-life care inclusion criteria is not met).

c This includes emergency, urgent or critical care.

d Where measures are not explicitly described as a PROM or PREM within a study, inclusion may be justified if they are self-reported or completed by a proxy (caregiver or professional) and do not meet exclusion criteria.

All citations were transferred to Endnote x9, duplicates were removed and all titles and abstracts were screened against the inclusion criteria by FH. A total of 10% of retrieved titles/abstracts were screened by ME, RG, and JH with a congruence of 95.6% achieved between screeners. Where differences were identified in screening, these were further discussed, and agreement was reach. A full-text review was conducted by FH on all papers that passed this initial screening with a calibration exercise conducted as recommended by Mak and Thomas.^20^ This is described as a process where 5–10% of papers are selected for independent screening. Where the level of agreement falls below 90%, points of disagreement are discussed and further calibration is required.^20^ A 7 per cent sample of papers that passed the initial screening received a full-text screening by second reviewers, CN and RG. A congruence of 100% was reached within the team.

### Data extraction

Relevant data from the articles were extracted by FH using Microsoft Excel. This included: authors, title, aim, study design, country, type of setting, sample, definition of frailty used and PROM(s) or PREM(s) found within the study. A second process of data extraction specific to PROMs/PREMs identified from the articles was conducted for the following information: PROM/PREMs name, concepts and sub-concepts, development sample; scaling method(s), number of items, recall period, evidence of testing for validity and/or reliability with older people with frailty, availability of a proxy measure, evidence of measuring the needs of carers, and ability to individualise the tool to include tailored symptom or concern items.

### Data synthesis

To enable the critical assessment of the conceptual properties of the included PROMs and PREMs, their content and scope were evaluated against a palliative care need framework^15^ and the component subdomains are detailed in Supplementary Data File 3. The assessment was discussed and agreed upon by the authors. A collated summary of results is found in the results below.

## Results

### Identification of studies

**Figure 1.**
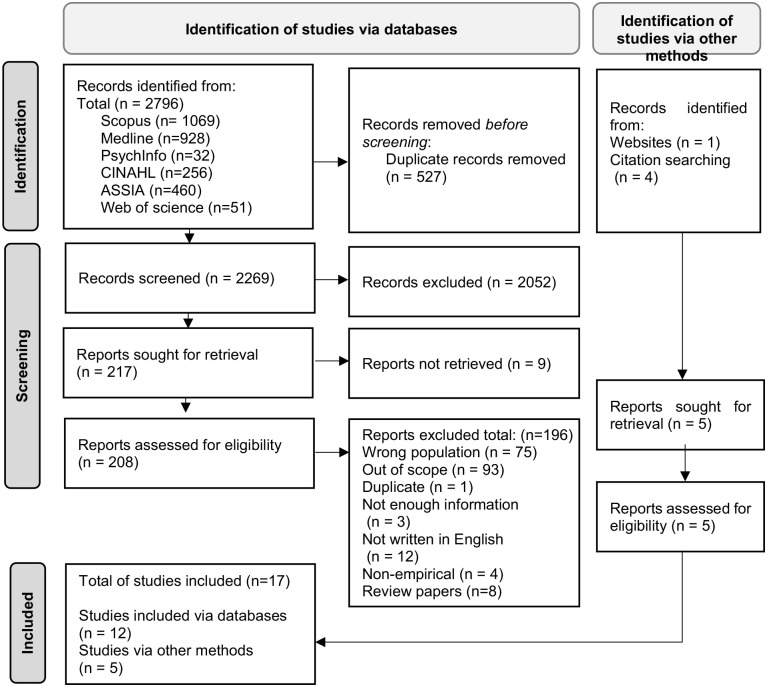
Prisma flow diagram.

Following the removal of duplicates, 2269 citations were screened of which 207 were included in a full-text screening (Figure 1). Of these, 195 were excluded and a total of 17 papers were included. These were predominately cross-sectional studies mainly from high-income countries with a focus on community-based long-term care (Table 2). Twelve peer-reviewed papers were identified within the database searches. A further five papers were found through hand searching. Of these five, four journal papers were obtained through back-searching citations^21–24^ and one non-peer-reviewed report was found through a hand search of grey literature.^25^ Out of the 17 included papers, 3 formed part of the same larger research study.^21,26,27^

**Table 2.** Details of results.

Paper	Paper’s AIM	Study design	Setting	Country	Definition of frailty	N. participants	N. with frailty	Tool
Borkenhagen et al.^28^	To assess whether the level of self-reported symptoms predicts unplanned readmission or emergency department (ED) care within 30 days within a population of elderly adults enrolled in a posthospitalisation program	Retrospective cohort	Integrated care	USA	Own definition	230	230	ESAS
Chochinov et al.^22^	To identify four non-cancer populations that may benefit from palliative care and patterns of dignity within these group	Prospective cohort	Acute and community care	Canada	Own definition	404	102	Patient dignity Inventory
de Nooijer et al.^29^	To describe palliative symptoms and needs of older people with frailty on hospital discharge to home.	Cross-sectional	Acute	Belgium	Operational (Rockwood Clinical Frailty Scale)	37	37	IPOS and ICECAP-SCM
Geessink et al.^30^	To investigate self-perceived and health-related QOL in older adults with frailty living in the community with and without a cancer diagnosis cross-sectionally	Cross-sectional	population level	Netherlands	Operational (Rockwood Frailty Index)	7493	7493 (6736 frailty without cancer, 751 frailty with cancer)	EuroQol-5D
Hall et al.^23^	To investigate the concept of quality of life to older adults receiving dialysis and the extent to which existing quality of life instruments reflect this.	Cross sectional	Acute	USA	Operational The FRAIL questionnaire	12	6	KDQOL_36, WHOQOL-OLD
Hoogendijk et al.^21^	To describe the met and unmet care needs of older people with frailty and explore this in association with socio-demographic and health-related characteristics.	Cross-sectional	Community	Netherlands	Operational (Combined Gobbens AND PRISMA-7)	1137	1137	Camberwell Assessment of Need for the Elderly (CANE)
Makai, et al.^31^	i) To evaluate the cost-effectiveness of an integrated care model for frail elderly, ii) to compare two different measures used to evaluate wellbeing	Cross-sectional	Integrated care	Netherlands	Operational (Groningen Frailty Indicator)	335	335	ICECAP-O, EQ-5D
Nicholson, et al.^32^	To describe the characteristics, symptoms, and concerns of older people with multi-morbidity/frailty in receipt of a community palliative care service; and compare the population with people receiving standard community-based specialist palliative care.	Cross-sectional	Specialist palliative	UK	Operational (Rockwood Frailty Index)	2069	815	IPOS
Pollack et al.^33^	To assess symptoms of older post-ICU patients and identify whether those with frailty have the greatest palliative care needs.	Prospective cohort	Acute and community care	USA	Operational (Fried Phenotype)	125	107	ESAS
Ratcliffe et al.^34^	To compare the measurement properties of 2 PROMs and their proxy versions in a population of older people with frailty in residential care, following hospital discharge for a hip fracture.	Prospective cohort	Long term care	Australia	Own definition	240	240	EQ-5D-5L, CEQ-5D-5L, DEMQOL-U, DEMQOL-Proxy-U
Sandgren et al.^35^	To assess the quality of life of older adults with frailty living in nursing homes and identify any differences in gender and age groups	Cross-sectional	Long term care	Sweden	Own definition	78	78	WHOQOL-OLD and WHOQOL-BREF
Seers et al.^25^	To explore the feasibility of the MYCaW® tool within a community service for older adults with frailty and identify the specific needs of people living with mild, moderate and severe frailty	Cross-sectional	Community	UK	Operational (Rockwood Clinical Frailty Scale)	310	310	MyCaW
Serrono et al.^36^	To examine the impact of biological frailty syndrome on quality of life in a population of older adults without severe cognitive decline living in nursing homes and identify the differences between men and women.	Cross-sectional	Long term care	Spain	Operational (Fried Phenotype)	281	151	SF-12
Van Leeuwen, et al.^26^	To compare the test-retest reliability, construct validity, and responsiveness of three PROMs in a population of older people with frailty living in the community.	Cross sectional	Community	Netherlands	Operational (Combined Gobbens AND PRISMA-7)	190	190	ASCOT SCT4, EQ-5D-3L, ICECAP-O
Van Leeuwen, et al.^27^	To translate and perform a cross-cultural validation of the ASCOT SCT-4 from English to Dutch for use in the Netherlands	Mixed method/stage design	Community	Netherlands	Operational (Combined Gobbens AND PRISMA-7)	190	190	ASCOT SCT4, EQ-5D-3L, ICECAP-O
Van Loon et al.^24^	To explore the impact of dialysis or MCM on the quality of life in older patients	Longitudinal	Acute	Netherlands	Operational Geriatric Assessment	281	226	EQ-5D-3L
Varela et al.^37^	To describe the quality of life of older people with frailty in the care of the Healthcare Centre of referrals, São Paulo, Brazil.	Cross-sectional	Community	Brazil	Own definition	122	122	WHOQOL-BREF, WHOQOLOLD, SF-36

### Characteristics of studies

Studies identified were conducted in eight high-income countries and one upper middle-income country. These included the Netherlands,^21,24,26,27,30,31^ USA,^23,28,33^ UK,^25,32^ Australia,^34^ Belgium,^29^ Canada,^22^ Spain,^36^ Sweden^35^ and Brazil.^37^

Study settings were described as community care,^21,25–27,37^ integrated care,^28,31^ long term care,^34–36^ acute care,^23,24,29^ mixed community and acute settings^22,33^ specialist palliative community care^32^ and population level study.^30^

### Conceptualisation of frailty

The definition of frailty varied among the studies. Of the included studies, 5 studies used their own definition of frailty but did not attempt to measure severity,^22,28,34,35,37^ and the remaining 12 used established operational definitions. These included: Fried’s Phenotype definition^38^; Groningen Frailty indicator^39^; Gobbens definition and PRISMA-7 questionnaire^40^; Rockwood Clinical Frailty Scale^41^; Rockwood Frailty Index^38^; geriatric assessment^42^; The FRAIL questionnaire^43^ No operational definition was used more than three times across the papers, evidencing the lack of a shared operational definition of frailty in wide usage. See Supplemental Data File 4 for further details.

### Identification of PROMS and PREMs

Of the 17 studies, 5 were related to the validation or implementation of a measure.^25–27,31,34^ The remaining 12 used a measure to identify needs and/or experience of those with frailty. Of the five validation and implementation studies, three compared the validity of a PROM against other PROMs,^26,31,34^ one examined the feasibility of implementing a PROM within a frail population^25^ and one examined the cross-cultural translation of a PROM from English to Dutch.^27^ From the 17 papers included within the review, 18 multi-dimensional measures were identified: 17 PROMs and 1 PROM with PREM elements (MyCaw); no PREMs were found (Table 3). One of the included papers, de Nooijer et al.,^29^ also included one item from a PREM (Views on Care) but this measure was not included within the synthesis of this review as it did not meet the inclusion criteria because it did not include the whole measure. Of the 18 PROMs, 2 were the proxy version of the self-reported measure which were also included within the review (CEQ-5D-5L and DEMQOL-Proxy-U). The authors were unable to source copies of two measures (CEQ-5D-5L, SF12) which impacted upon the data synthesis of these measures. No measure was identified more than three times across different papers and nine were only identified once. The recall period used in the PROMs varied. Where the recall period was stated, this ranged from contemporaneous to 6 months. It was not stated in two measures (AscotSC4, CANE adapted) and it was unclear how it was opertwotionalised in one (SF12). The mean and median number of items per measure was 16.7 and 12.5 items, with a range from 5 (EQ_5D_3L; EQ_5D_5L; MyCaw) to 36 (SF-36 and KDQOUL). Three of the found measures (ESAS, IPOS, MyCAW) could be individualised to include personalised symptom or concern items. IPOS was the only measure that had any specific items which addressed the needs of the caregiver within the patient version.

**Table 3.** Summary of measures.

Measure abb. *Full name*	Description	Overarching concept *(Original target population*)	Scaling method	No. items	Recall period	Evidence of validity reliability with frailty population	Papers (*n*)
CEQ-5D-5L *Caregiver-EuroQOL five-dimensional questionnaire-5L*	PROXY version of EQ-5D – Used to measure value health-related QOL to calculate quality-adjusted life years for cost analysis.	Health-related quality of life *(Generic measure)*	Ordinal 5 category scale (‘I have no problem’ to ‘I am unable’	5	Current ‘*TODAY*’	Convergent validity with proxy measure: Spearman correlation (Ratcliffe, 2017); Known-group validity: Kruskal-Wallis test (Ratcliffe, 2017)	Ratcliffe et al. (2017) (1)
EQ-5D-3L *EuroQOL five dimensional questionnaire-3L*	Used to measure value health-related QOL to calculate quality-adjusted life years for cost analysis.	Health-related quality of life *(Generic measure)*	Ordinal 3-category scale (no problems to extreme problems)	5	Current *‘TODAY*’	Construct validity: moderate correlation Cohen’s convention with EQ-5D-3L and ICECAP-O (Van Leeuwen 2015a); test re-test: ICC > 0.70 (Van Leeuwen 2015a); responsiveness: Similar to ICECAP-O (Van Leeuwen 2015a)	Van Leeuwen, et al. (2015a), Van Leeuwen et al. (2015b), Makai et al. (2015) and Van Loon et. al (2019) (3)
EQ-5D-5L *EuroQOL five dimensional questionnaire-5L*	Used to measure value health-related QOL to calculate quality-adjusted life years for cost analysis.	Health-related quality of life *(Generic measure)*	Ordinal 5 category scale (‘I have no problem . . .’ to ‘I am unable to . . .’	5	Current ‘*TODAY*’	Convergent validity with proxy measure: Spearman correlation (Ratcliffe, 2017); Known-group validity: Kruskal-Wallis test (Ratcliffe, 2017)	Ratcliffe et al. (2017) and Geessink et al. (2017) (2)
SF-12 *RAND Short form 12*	Generic Quality of life measure - 12 items: used for assessing the cost-effectiveness of health care interventions	Health-related quality of life *(Generic measure)*	Binary (Yes/No); Ordinal category 3, 5 and 6 point scale (‘Yes, Limited a lot’ to ‘No, Not Limited at all’	12	‘the past 4 weeks’		Serrono et al. (2017) (1)
SF-36 *RAND Short form 36*	Generic Quality of life measure – 36 items: used for assessing the cost-effectiveness of health care interventions	Health-related quality of life *(Generic measure)*	Binary (Yes/No); Ordinal category 3, 5 and 6 point scale (‘Yes, Limited a lot’ to ‘No, Not Limited at all’ Likert (Definitely true to Definitely false)	36	‘*Compared to one year ago . . .*’ and ‘*the past 4 weeks*’		Varela et al. (2015) (1)
WHOQOL-BREF *World Health Organisation Quality of Life-BREF*	WHO Quality of life measure – Shorter than original 100-item measure	Health-related quality of life *(Generic measure)*	Five-level numerical/written scale	26	Weeks ‘*in the last two weeks*’		Varela et al. (2015) and Sandgren et al. (2021) (2)
KDQOL 36 *Kidney Disease Quality of Life 36*	Quality of life measure – 36 items developed for patients with kidney disease. Part of the RAND group of measures. First 12 items same as SF12	Health-related quality of life (*Patients with Kidney Disease*)	Binary (Yes/No Ordinal category 3, 5, 6 and 10 (‘Yes, Limited a lot’ to ‘No, Not Limited at all’) Likert (Definitely true to Definitely false)	36	*Weeks* ‘*the past 4 weeks*’		Hall et al. (2020) (1)
WHOQOLOLD *World Health Organisation Quality of Life OLD*	The WHOQOL-OLD is a multi-dimensional measure of quality of life in older persons and comprises 24 items divided into six facets or subscales of four items each	Health-Related Quality of Life (*Older people*)	Ordinal five category scale from (‘1 = Not at all’ to ‘5 = An extreme amount’)	24	Weeks ‘*in the last two weeks*’		Varela et al. (2015), Sandgren et al. (2021), Hall et al. (2020) (3)
DEMQOL-Proxy-U *Dementia Quality of Life-Proxy*	Proxy version of DEMQOL health-related quality of life of people with dementia.	Health-related quality of life (*People with Dementia*)	Ordinal four-category scale (‘a lot’ to ‘not at all’)	32	Week ‘*In the last week*’	Convergent validity with proxy measure: Spearman correlation (Ratcliffe, 2017); Known-group validity: Kruskal-Wallis test (Ratcliffe, 2017)	Ratcliffe et al. (2017) (1)
DEMQOL-U *Dementia Quality of Life*	Developed to measure health-related quality of life of people with dementia.	Health-related quality of life (*People with Dementia*)	Ordinal four-category scale (‘a lot’ to ‘not at all’)	28	Week ‘*In the last week*’	Convergent validity with proxy measure: Spearman correlation (Ratcliffe, 2017); Known-group validity: Kruskal-Wallis test (Ratcliffe, 2017)	Ratcliffe et al. (2017) (1)
ICECAP-O *ICEpop CAPability measure for older people*	Preference-based measure, based on a capability approach, defining well-being in relation to an individual’s ability to ‘do and be’ the things that are important in life	Quality of life (*People aged +65*)	Ordinal 4 category scale (‘I can . . .’ to ‘I am unable . . .’)	5	Current ‘*at the moment*’	Construct validity: moderate correlation Cohen’s convention with EQ-5D-3L and ICECAP-O (Van Leeuwen 2015a); test re-test: ICC > 0.70 (Van Leeuwen 2015a); responsiveness: weak correlation – however similar to EQ-5D-3L (Van Leeuwen 2015a)	Van Leeuwen et al. (2015a) and Makai et al. (2015) (2)
ICECAP-SCM *ICEpop CAPability Supportive Care measure*	Preference-based measure, based on a capability approach, defining well-being in relation to an individual’s ability to ‘do and be’ the things that are important in life	Quality of life (*older people: in the general population; residential care and palliative care*)	Ordinal 4 category scale (‘I can . . .’ to ‘I am unable . . .’)	7	Current ‘*at the moment*’		De Nooijer et al. (2022) (1)
ASCOT SCT4 *Adult Social Care outcome toolkit: Four level Self Complete questionnaire*	Developed to measure social care-related quality of life covering personal care; security, social participation; occupation, dignity; independence/control	social care-related quality of life (*Adults in receipt of social care*)	Ordinal 4 category scale (‘I’m able to’ to ‘I don’t do’)	9	Not stated	Content validity: Cognitive interviews (Van Leeuwen 2015b) Cross-cultural content): compared English and Dutch version validity (Van Leeuwen 2015b). Construct validity: moderate correlation Cohen’s convention with SF12, EQ-5D-3L and ICECAP-O (Van Leeuwen 2015a); test re-test: ICC > 0.70 (Van Leeuwen 2015a); responsiveness: 0.34 weak correlation (Van Leeuwen 2015a)	Van Leeuwen, et al. (2015a) and Van Leeuwen, et al. (2015b) (2)
CANE-adapted *Camberwell Assessment of Need for the Elderly-Adapted (includes 13 individually validated items from original measure)*	Shorter form of full CANE was developed to measure the met and unmet needs of older people with mental disorders by recording staff, carer, and patient views, especially for people with mental disorders	Symptoms and needs (*Older people in social and clinical settings*)	Ordinal four-category scale	13	Not stated		Hoogendijk et al. (2016) (1)
ESAS *Edmonton Symptom Assessment Scale*	Used to rate the intensity of nine common symptoms experienced by cancer patients, including pain, tiredness, nausea, depression, anxiety, drowsiness, appetite, well-being and shortness of breath.	Symptoms and needs (*Palliative cancer patients*)	Ordinal 11-category scale (no problems to extreme problems)	10	Current ‘*how you feel now*’		Pollack et al. (2017) and Borkenhagen et al. (2018) (2)
IPOS *Integrated Palliative Outcome scale*	Tool to measure Palliative care needs of patients and their families. Developed from the POS	Symptoms and needs (*Palliative patients*)	Ordinal 5 category (‘Not at all’ to ‘overwhelmingly’ and free text	17	Days or weeks ‘*past 3 days*’ ‘*over the past week*’		Nicholson et al. (2018) and De Nooijer et al. (2022) (2)
MYCaW *Measure Yourself Concerns and Wellbeing*	A person-specific PROM measures two individualised concerns or problems and general well-being. Also, two free text experience-related questions	Symptoms and needs (*Holistic cancer support settings*)	Free text and Ordinal seven category (0 = *Not bothering me at all*; 6 = *Bothers me greatly*)	3	Current ‘*now*’		Seers et al. (2022) (1)
PDI *Patient Dignity Inventory*	25 items questionnaire to give clinicians a broad overview or ‘snapshot’ of how someone in their care is doing	Symptoms and needs (Palliative cancer patients)	Ordinal five category scale from (1 = *Not a problem*; 5 = *overwhelming problem*)	25	Days ‘*within the last few days*’		Chochinov et al. 2016 (1)

Overarching concepts of the measures include health-related quality of life (*n* = 10), symptom and need (*n* = 5), quality of life (*n* = 2) and social care-related quality of life (*n* = 1). All the measures were developed within an adult population. Six of these were categorised as generic measures (not developed for a specific condition, group or need). The specific measures were developed for palliative and/or cancer patients (*n* = 4), older people (*n* = 3), older people with dementia (*n* = 3), those in receipt of social care (*n* = 1) and kidney disease patients (*n* = 1). Of all the measures, 11 were either proxy measures or available as proxy measures.

### Examining the extent of the validity of the measures

Seven out of the 18 measures had evidence that they had been tested for validity and/or reliability within the specific frailty population defined within their study (ASCOT Sc4; CEQ-5D-5L, DEMQOL-Proxy-U, DEMQOL-U, EQ-5D-3L, EQ-5D-5L, ICECAP-O), four of which were generic measures (CEQ-5D-5L, EQ-5D-3L, EQ-5D-5L). Evidence was found of construct validity (*n* = 7), internal consistency (*n* = 2), and content validity (ASCOT Sc4). No measure was developed specifically for a frailty population. Of the five measures which were originally developed for those with life-limiting conditions (ESAS, ICECAP-SCM, IPOS, MyCAW and PDI), none had evidence of validation studies within a frailty population.

### Examining the measure against domains of end-of-life need

The MyCAW measure, which was completely individualised, was considered to cover all domains, given that those completing the tool would be able to address the concerns that matter to them most. Physical, psychological and social domains had good coverage within the measures. The spiritual and practical domains, however, had less coverage, and therefore a further synthesis of how tools addressed these two domains is described. Excluding the individualised measure, just five measures covered all five domains (ICECAP_SCM, IPOS, PDI, WHOQOL-BREF, WHOQOL-OLD). The coverage of items within the domains varied between the measures.

### Physical, psychological and social domains

All measures had items within the physical, psychological, and social domains, except for ESAS which covered physical and psychological domains only. Notably, the ESAS measure was one of the few measures that allowed for individualised symptom or concern items to be added, which may allow for the inclusion of other domains of need. Out of these three domains, the physical domain had the largest item coverage across all combined measures, while the social domain had the least.

### Spiritual domain

Excluding MyCAW, six measures had coverage within the spiritual domain: ICECAP-O, ICECAP-SCM, IPOS, PDI, WHOQOL-BREF, WHOQOL-OLD. The extent of the coverage of items within this domain, however, varied between the measures. IPOS, for example, only had 1 item within the spiritual domain (out of 17 items), whereas the WHOQOL-OLD had 7 spiritual items (out of 24). Most of the items across the six measures fell under the subdomain spiritual – *Questioning/distress of loss of future or hope*. Only ICECAP-SCM and PDI had items that were deemed to fall under the spiritual subdomain – *Seeking and or expressing connectedness to existence*.

### Practical domain

Excluding MyCAW, nine measures addressed the practical domain: ASCOT, CANE (adapted), DEMQOL, DEMQOL-PROXY, ICECAP-SCM, IPOS, KDQOL, PDI and WHOQOL-BREF. Items across these nine measures covered the practical subdomain, *Information, and financial needs*, reasonably well. The subdomains, *Access to and receipt of care, Environmental care needs, and care planning and planning for the futur*e, had less coverage. The subdomain of *care planning, planning for the future*, however, was only covered by ICECAP-SCM (Table 4).

**Table 4.** Item coverage in domains of palliative care framework.

Measure	Developed for life-limiting condition	Proxy version available	Can it be individualised	Needs of carer	Item coverage in domains of palliative care framework (Nicholson et al., 2023)^2^				
					Physical	Social	Psychological	Spiritual	Practical
ASCOT SCT4		X	*	ꬷ	X	X	X		X
CANE-adapted		‡		¶	X	X	X		X
CEQ-5D-5L		X			X	X	X		
DEMQOL-Proxy-U		X			X	X	X		X
DEMQOL-U		X			X	X	X		X
ESAS	X	X	X		X		X		
EQ-5D-3L		X			X	X	X		
EQ-5D-5L		X			X	X	X		
ICECAP-O			X		X	X	X	X	
ICECAP-SCM	X				X	X	X	X	X
IPOS	X	X	X	X	X	X	X	X	X
KDQOL-36	X				X	X	X		X
MYCaW	X	X	X		X	X	X	X	X
PDI	X				X	X	X	X	X
SF-12		X			X	X	X		
SF-36		X			X	X	X		
WHOQOL-BREF					X	X	X	X	X
WHOQOLOLD					X	X	X	X	X

* Free text option in proxy version.

ꬷ Separate measure available for carers.

‡ Patient AND professional.

¶ Found in full measure.

## Discussion

### Main findings

To our knowledge, this is the first review to assess how the multi-dimensional needs experienced by those with severe frailty nearing the end of life are conceptualised within existing PROMs and PREMs, to examine the extent of their validation with this patient group, and to determine the context of their use.

The studies found within this review were predominantly from high-income countries. There is some evidence that the prevalence of frailty is higher within upper-middle-income countries compared to high-income countries, suggesting that the findings of this review should have global relevance.^44^ The varied conceptualisations of frailty found within the papers in the review support the wider literature, indicating a lack of an international consensus on operational ways to identify frailty. This continues to be problematic in relation to making international comparisons.^1^ The settings within which the studies were conducted are largely described as community care or long-term care, with only one study conducted within a specialist palliative setting.^32^ This perhaps reflects other evidence that shows that very few older adults with frailty receive end-of-life provision from specialist palliative care services.^45^ The degree of validity and reliability of the found measures with those with frailty was limited, with scarce evidence of face validation. Face validity (often termed subjective validation) is concerned with the meaningfulness, appropriateness and relevance to the user (in this case, the patient or service user)^46^ (p. 5). The limited evidence of face validity within this specific population raises the question of whether a given measure and its included outcomes actually matter to this group of individuals (in contrast to what the researcher or professional perceive to matter).

This is a population with a wide variety of possible need. The fact that 18 measures (9 of which were identified only once) were identified in this review reflects not only the challenge of measure selection for this population but also the absence of a measure developed specifically for this group. When the found measures were evaluated against the palliative care conceptual framework, and the coverage within each of the subdomains was considered, no measure addressed all the components. Table 5 illustrates this lack of coverage. Particularly worthy of note is the lack of measures addressing practical and spiritual domains where, for example, the subdomain *Informational Needs and Care Planning Needs* was hardly covered. This, however, was particularly difficult to apply to MyCAW which was unique compared to the other measures in comprising of free text items that related to individualised concerns or problems. Originally developed for cancer care patients, MyCAW is possibly the only measure that allows it to be tailored to the individual while still producing quantifiable metrics. MyCAW was also the only measure to include any experience items. This limited evidence of PREMs was also noted by Schick-Makaroff et al.,^47^ in a similar population who found their use was driven by evidencing interventions, rather than by assessing the needs and experiences of the individual. When comparing the findings from this scoping review with wider literature, two key issues emerge: i) whether items within measures under the physical domain are all applicable to those with frailty (particularly those measures developed for single life-limiting conditions), and ii) whether there are gaps in measures used to address the needs that may matter most to this group (i.e., within the practical and spiritual domains). Nicholson et al.,^2^ observed that expressed physical needs were often the least reported by this group, in contrast to the practical and spiritual domains that had the most unmet needs. This is possibly because healthcare providers are most equipped and trained to meet physical need compared to other domains. Others suggest the needs of those with frailty are more likely to be framed around the normalisation of symptoms; and where the concept of ‘*good health*’ is less of a priority to achieve wellbeing.^48,49^ This may be considered in relation to concepts of coping and adjustment but also emphasises the multifactorial and dynamic nature of frailty, where what matters most to individuals moves beyond physical symptoms and changes both over time and in relation to other factors.

**Table 5.** Item coverage in subdomains of spiritual and practical need within the palliative care framework.

Measure (total number of items )	ASCOT (*n* = 9)	CANE-adapt (*n* = 13)	DEMQOL (*n* = 28)	DEMQOL-proxy (*n* = 32)	IceCAP-O (*n* = 5)	IceCAP-SCM (*n* = 7)	IPOS (*n* = 17)	KDQOL (*n* = 36)	PDI (*n* = 25)	WHOQOL-BREF (*n* = 26)	WHOQOL-old (*n* = 24)
Subdomains of spiritual need											
Questioning/distress of loss of hope or loss of future					1		1		3	1	5
Seeking and or expressing connectedness to existence						1			1		
Subdomains of practical need											
Environmental care needs, housing, aids, or adaption	2	4	2	2			1	2		4	
Information and or financial needs		1					1			2	
Care planning, planning for the future						1					
Access to and receipt of individualised care						1			1	1	

There was good evidence of the use of proxy measures or measures that had proxy versions found in this review. This is particularly relevant to the populations of older people with severe frailty due to the possibility of increased fatigue and physical/cognitive deterioration. A lack of coverage of caregiver needs was, however, noted. The role of the unpaid/family caregiver in end-of-life care has long been recognised as important, but it is only in recent years that attention has been focused on their own needs.^50,51^ The increased use of measures such as the Carer Support Needs Assessment Tool (*CSNAT*) and *ASCOT Carers* indicates a positive change, reflecting a shift in attempts to capture and address carers' needs in their own right.^52,53^ Much of our understanding of caregiver needs in end-of-life care has, however, also been developed from work with carers of those with cancer and does not necessarily reflect the needs of other caregivers.^50^ The needs of those with frailty and their caregivers are arguably intertwined, particularly where the caregiver themselves may be older and have their own health conditions or have caring responsibilities for younger family members.^54,55^ These needs, however, rarely seem to be examined as a patient-caregiver dyad. The implementation of measures that are able to reflect this dyad is not only important for addressing the unmet needs of those with frailty but also their caregivers.

### Implications for practice, policy and research

Access to end-of-life care is not equal for everyone^10^ and those with frailty are less likely to receive specialist end-of-life care than other groups with single life-limiting conditions.^14^ A move towards a public health concept of care which offers a needs-based approach within an assessment, may be more appropriate for tailoring individual care for older people with frailty.^3^ This contrasts with a traditional disease-specific model driven by prognostic markers which is particularly problematic for addressing the needs of this group.^19,56^ Measures in use must also be able to hold the tension between living *with* frailty and dying *as well as possible*, in addition to the complexity and uncertainty faced by these individuals and their caregivers.^56,57^ Any tailored PROMs and PREMs for this group need to be implemented beyond specialist palliative care settings, and used by generalist health and social providers who typically care for this group.

This review has highlighted three gaps within which further work may improve the development and use of PROMs and PREMs with older people with frailty nearing the end of life: i) A need for a better understanding of how existing measures are used at an individual level and how there are embedded in practice. ii) A consideration of how PROMs and PREMs can be used together and the practical factors that may influence how this is achieved. iii) Measures used in practice should be tailored to the needs of this group, with future research focusing on how existing PROMs and PREMs could be made fit for purpose, with adaption and validation.

One of the appeals of PROMs and PREMs to service providers is their capacity to generate quantifiable data from something subjective. It is what has made them useful in evidencing the quality of care in a value-based model of care.^58^ What appears lacking from the evidence is how PROMs or PREMs are being used within practice with this group. This, in turn, raises questions related to who gains the most from their use. Can the use of PROMs and PREMs give older people with frailty and caregivers a voice where the act of completing a measure opens opportunities for practitioners to listen, hear and respond to what matters most? The answer to this must partly lie in gaining a better understanding of their use. The example of the patient-completed clinical tool, SNAP, is useful here. It is considered by its developers to be a tool to support person-centred care rather than an outcome measure.^59^ The emphasis of its use lies, not as a tick box exercise, but within an identification of needs, discussion and response cycle which can flex to the individualised needs and values of the user rather than as a means to provide evidence of quality of care. Apart from the ethical implications of such practice, to what extent are the needs that we capture and measure guided by the patient or from the tool itself? If the latter is the case, needs which fall outside the parameters of a tool are at risk of not being identified and are ultimately at risk of not being addressed. Thus, questions for future research are: i) how are these measures being implemented within practice to deliver care that addresses the needs that matter at an individual level and ii) how these converge with organisational and population level agendas which benefit from their use.

The inclusion of a practical domain in the framework of need is significant for both care organisations, who want to evidence the impact of their care, and those with frailty, who are more likely to be dependent on others to support autonomous living. For a group which has fluctuations and more gradual declines in physical health towards the end of life compared to other groups,^60^ the beneficial impact of the care that organisations deliver may be harder to measure using symptom-focused tools alone. For the older person with frailty, what an older person might express as a need may not be recognised as such by care providers. Both recognising and treating care providers’ values, for example, access to individualised care, as practical needs would mean that these needs become directly dependent on the parameters of services to address them, rather than indirectly being tied to care providers’ values. If care providers are not addressing this population’s practical needs, it suggests an adaptation of their services is required in order to apply their values of care more meaningfully. In this instance, the use of PREMs alongside PROMs may be useful. Schick-Makaroff et al.,^47^ argue that using PREMs and PROMs together may provide an opportunity to inform and improve care and provide quantifiable evidence of need, without which commissioning bodies do not have the urgency to respond. Others, however, suggest more consideration is required to how PREMs are used with attention given to some of the practical barriers that might get in the way of making a difference to people’s care, and to the ethical implications of who and how these are collected.^61^ In a landscape where services are increasingly stretched, identifying and addressing practical needs at an individual level could have some of the biggest impacts on quality of life for an individual with frailty. The use of measures that incorporate experience and outcomes, particularly within this practical domain, may ensure that professionals and providers are able to take notice and address what matters most to those with frailty.

Determining the most appropriate PROMs or PREM suitable for a task is always challenging, particularly for this group who have diverse health conditions and needs, and where there is a lack of tools developed for them. The evidence surrounding the use of PROMs and PREMs within end-of-life care is growing.^9,10^ Numerous tools have been developed within end-of-life care, with Harding et al.,^62^ identifying approximately 100 outcome tools, although most were found to be cited fewer than 10 times. Rather than creating new tools, efforts should focus on how existing ones might be adapted.^62^ An example of this approach can be seen with the development of the IPOS-Dem PROM, which has been adapted from the IPOS to address the particular end-of-life needs of those with dementia within a care home setting.^63^ Although not developed for groups defined by frailty, this proxy measure works towards addressing the conceptual disparity between the end-of-life needs of those with cancer (with which the original IPOS and its predecessor POS were developed) and other groups. The recently developed PREM consideRATE, designed to measure serious illness experience, was indicated in a review of PREMs suitable for hospitalised patients with palliative care needs.^64,65^ This may be of merit for use with older people with frailty. Virdun et al.,^64^ emphasised that while the selection of the PREMs is important, they also highlight the need for greater attention on how data is captured and used to inform understanding of need within the populations that they are being used.

### Strengths and limitations

Initial results, without date limits, were found to have little specificity to the research question, hence the search was limited from 1st January 2012 to 27th April 2023. This parameter was chosen to reflect the increasing prominence of the term frailty over recent years. This decision means that it is a possibility that PROMs and PREMs developed or used before this date were missed. However, it was envisaged if a measure were established earlier, it would also be found in later papers. The majority of the screening of the review was undertaken by the lead author. Methods to mitigate bias are described in the design section. As part of inclusion criteria within this review, papers had to be written in English, due to a lack of resources to translate papers. Only one paper came from a middle-income country and the remaining were from high-income countries. This may reflect that frailty, as a syndrome, may be more recognised within high-income countries.

No frailty definition or used proxy definitions were imposed upon the studies as part of the inclusion/exclusion criteria in this review. Instead, it accepted the authors’ definition of frailty, and a similar approach was used by others.^14^ This was an attempt to ensure that the sample was clinically distinct from older people without frailty and in recognition that frailty as a concept has evolved, resulting in the adoption of different approaches to measuring and/or defining it. This decision had some limitations. Firstly, five studies within this review used their own definitions of frailty within their sample without attempting to measure severity.^22,28,34,35,37^ This highlights the need for future studies to be explicit regarding the method of defining and identifying frailty within their samples and how it is reported. This is also recommended by Hall et al.^19^ Conversely, this inclusion criteria may have excluded measures that may be relevant to this group. IPOS-Dem and SNAP may be considered as examples of this.^59,63^ It would be prudent to acknowledge the potential suitability of such measures, alongside others found to be used with this group, which exist outside the scope of this study.

## Conclusions

Multi-dimensional self-reported outcome measures are an important component in delivering quality care to older people with severe frailty. This review has highlighted a lack of evidence of validity in the currently available measures with this group; gaps in their coverage, particularly within the practical and spiritual domains; as well as very few identified PREMs. Existing measures have not been designed to address what we know about the needs of those with severe frailty. Rather than developing a new measure, which can be both costly and timely, future work should focus on how existing measures are currently used within practice and how these could be adapted and validated to be made more fit for purpose.

## Supplemental Material

sj-pdf-1-pmj-10.1177_02692163231223089 – Supplemental material for Understanding the extent to which PROMs and PREMs used with older people with severe frailty capture their multidimensional needs: A scoping review Click here for additional data file. Supplemental material, sj-pdf-1-pmj-10.1177_02692163231223089 for Understanding the extent to which PROMs and PREMs used with older people with severe frailty capture their multidimensional needs: A scoping review by Faith D Howard, Richard Green, Jenny Harris, Joy Ross and Caroline Nicholson in Palliative Medicine

sj-pdf-2-pmj-10.1177_02692163231223089 – Supplemental material for Understanding the extent to which PROMs and PREMs used with older people with severe frailty capture their multidimensional needs: A scoping review Click here for additional data file. Supplemental material, sj-pdf-2-pmj-10.1177_02692163231223089 for Understanding the extent to which PROMs and PREMs used with older people with severe frailty capture their multidimensional needs: A scoping review by Faith D Howard, Richard Green, Jenny Harris, Joy Ross and Caroline Nicholson in Palliative Medicine

sj-pdf-3-pmj-10.1177_02692163231223089 – Supplemental material for Understanding the extent to which PROMs and PREMs used with older people with severe frailty capture their multidimensional needs: A scoping review Click here for additional data file. Supplemental material, sj-pdf-3-pmj-10.1177_02692163231223089 for Understanding the extent to which PROMs and PREMs used with older people with severe frailty capture their multidimensional needs: A scoping review by Faith D Howard, Richard Green, Jenny Harris, Joy Ross and Caroline Nicholson in Palliative Medicine
