# Supplementary material for: Understanding the extent to which PROMs and PREMs used with older people with severe frailty capture their multidimensional needs: A scoping review
Source: Palliat Med. 2024 Jan 24;38(2):184–99. doi: 10.1177/02692163231223089 (PMC10865766; doi:10.1177/02692163231223089)
Supplement: sj-pdf-2-pmj-10.1177_02692163231223089 – Supplemental material for Understanding the extent to which PROMs and PREMs used with older people with severe frailty capture their multidimensional needs: A scoping review [file sj-pdf-2-pmj-10.1177_02692163231223089.pdf]

Supplementary Data File 3: Framework of palliative care need

Need domain	Definition
Physical	Any need arising from a biological cause related to:<br>Symptom burden or treatment<br>Decline in physical function and core activities related to activities of daily living.
Social	Any need relating to social roles and/or functioning.<br>*Relational care needs*: related to being in connection with others, including family carers and care professionals.<br>*Social network needs*: related to both supporting and being supported by family, friends, and neighbourhoods.
Psychological	Any need relating to:<br>Anxiety, depression<br>Self-esteem, self-worth, and adjustment to a situation.
Spiritual	Needs that arise from distress or questioning the loss of hope of a future.<br>Needs that relate to the way a person seeks and expresses their connectedness to existence.
Practical	Any need related to a requirement for resource(s) to enable a person to manipulate their environment so that they can live as well as possible both now and in the future.<br>Environmental care needs, such as housing, aids, or adaptations.<br>Informational and/or financial needs.<br>Care planning, including planning for the future.<br>Access to and receipt of individualised care.

Subdomains definitions were developed by Nicholson et al., (2022) and agreed as relevant to the population for older adults with multimorbidity (including frailty) by a clinical reference group of palliative and geriatric clinicians.
