# Supplementary material for: Understanding the extent to which PROMs and PREMs used with older people with severe frailty capture their multidimensional needs: A scoping review
Source: Palliat Med. 2024 Jan 24;38(2):184–99. doi: 10.1177/02692163231223089 (PMC10865766; doi:10.1177/02692163231223089)
Supplement: sj-pdf-3-pmj-10.1177_02692163231223089 – Supplemental material for Understanding the extent to which PROMs and PREMs used with older people with severe frailty capture their multidimensional needs: A scoping review [file sj-pdf-3-pmj-10.1177_02692163231223089.pdf]

Supplementary Data File 4: Operational definitions of frailty use within found papers.

**Operational Definitions/ Country**	Fried Phenotype	Groningen Frailty Indicator (GFI)	Combined Gobbens AND PRISMA-7	Rockwood Clinical Frailty Scale	Rockwood Frailty Index	The FRAIL questionnaire	Geriatric Assessment
**Belgium**				De Nooijer et al. 2022			
**Netherlands**		Makai, et al., 2015;	Hoogendijk et al. 2016; Van Leeuwen, et al., 2015a; Van Leeuwen, et al., 2015b*		Geessink, et al., 2017		Van Loon et al, 2019
**Spain**	Serrono, et al., 2017						
**UK**				Seers et al 2022	Nicholson, et al., 2018		
**USA**	Pollack, et al., 2017					Hall et al., 2020	
**Total**	2	1	3	2	2	1	1

Definition	Description
Fried's Phenotype model	Based on 5 indicators: weight loss; self-reported exhaustion; weakness; slow walking speed and low physical activity. Individuals are categorised as either: robust (none of the indicators); pre-frail (1-2 indicators); or frail (3 or more indicators). It is a good predictor of frailty in non-disabled and can be used as a tool to indicate further assessment. It is criticised that it has no application for disabled groups. Based on signs and symptoms, it cannot be used to initiate interventions as it does not indicate a cause (1)
Rockwood - Frailty Index	Based on the cumulative deficit model developed by Rockwood and colleagues which identifies frailty as an accumulation of deficits associated with aging (2). The deficits can be grouped into four distinct sets: disease; function; laboratory results and symptoms or signs. Each deficit carries equal weight in this model. The higher number of deficits, the higher their frailty index.<br>It is gradable and can differentiate between severity of frailty and can be sensitive when identifying a change in an individual's condition(3). Requires a comprehensive clinical assessment.
Rockwood - Clinical Frailty Scale	A screening tool based on the cumulative deficit model. It is a 9-point ordinal scale, which is accompanied with pictorial and textual descriptions. It is relatively quick to use making it clinically feasible as it does not require a full clinical assessment, however it does not provide precise measurement(4).
Groningen Frailty Indicator (GFI)	A validated multidimensional self-reported questionnaire developed within the Netherlands. The higher the score indicates higher need, with a score of 4 indicating frailty. (5)
Gobbens, et al., (2010)	A conceptual definition which considers frailty as "a multidimensional and dynamic state that has adverse physical, psychological, and social outcomes"
PRISMA-7	Based on a phenotype model of frailty, it is a quick self-completed questionnaire comprised of 7 items which can be used as a screening tool for frailty. A score of 3 or more is considered to indicate frailty. (6)
The FRAIL questionnaire	Five item questionnaire completed by clinician addressing **F**atigue, **R**esistance, **A**mbulation, **I**llness and **L**oss of weight. (7)
Geriatric assessment	A multidisciplinary holistic assessment of the older adult which should result in a plan of care to address issues and concerns (8).

1. Cesari M, Gambassi G, Abellan van Kan G, Vellas B. The frailty phenotype and the frailty index: different instruments for different purposes. Age Ageing. 2014 Jan 1;43(1):10–2.
2. Mitnitski AB, Mogilner AJ, Rockwood K. Accumulation of Deficits as a Proxy Measure of Aging. The Scientific World JOURNAL. 2001;1:323–36.
3. Clegg A, Young J, Iliffe S, Rikkert MO, Rockwood K. Frailty in elderly people. The Lancet. 2013 Mar;381(9868):752–62.
4. Rockwood K. A global clinical measure of fitness and frailty in elderly people. Can Med Assoc J. 2005 Aug 30;173(5):489–95.

5. Peters LL, Boter H, Buskens E, Slaets JPJ. Measurement Properties of the Groningen Frailty Indicator in Home-Dwelling and Institutionalized Elderly People. J Am Med Dir Assoc. 2012 Jul;13(6):546–51.
6. British Geriatric Society. Introduction to frailty, Fit for Frailty Part 1. 2014.
7. Morley JE, Malmstrom TK, Miller DK. A simple frailty questionnaire (FRAIL) predicts outcomes in middle aged African Americans. J Nutr Health Aging. 2012 Aug 11;16(7):601–8.
8. Ellis G, Whitehead MA, Robinson D, O'Neill D, Langhorne P. Comprehensive geriatric assessment for older adults admitted to hospital: meta-analysis of randomised controlled trials. BMJ. 2011 Oct 27;343(oct27 1):d6553–d6553.
